# Magnetic Resonance Imaging-Based Monitoring of the Accumulation of Polyethylene Terephthalate Nanoplastics

**DOI:** 10.3390/molecules29184380

**Published:** 2024-09-14

**Authors:** Narmin Bashirova, Erik Butenschön, David Poppitz, Henrik Gaß, Marcus Halik, Doreen Dentel, Christoph Tegenkamp, Joerg Matysik, A. Alia

**Affiliations:** 1 Institute of Medical Physics and Biophysics, Leipzig University, D-04107 Leipzig, Germany; narmin.bashirova@medizin.uni-leipzig.de; 2 Institute of Analytical Chemistry, Leipzig University, D-04103 Leipzig, Germany; erik.butenschoen@uni-leipzig.de (E.B.); joerg.matysik@uni-leipzig.de (J.M.); 3 Institute of Chemical Technology, Leipzig University, D-04103 Leipzig, Germany; david.poppitz@uni-leipzig.de; 4 Organic Materials & Devices Institute of Polymer Materials, Friedrich-Alexander-University Erlangen-Nürnberg, D-91058 Erlangen, Germany; henrik.gass@fau.de (H.G.); marcus.halik@fau.de (M.H.); 5 Institut of Physics, Technical Chemnitz University, D-09126 Chemnitz, Germany; doreen.dentel@physik.tu-chemnitz.de (D.D.); christoph.tegenkamp@physik.tu-chemnitz.de (C.T.); 6 Leiden Institute of Chemistry, Leiden University, 2333 CC Leiden, The Netherlands

**Keywords:** polyethylene terephthalate, nanoplastics, µMRI, SPION, wheat

## Abstract

Polyethylene terephthalate (PET) is one of the most produced plastic materials in the world. The emergence of microplastics and nanoplastics (MPs/NPs) as a significant environmental contaminant has become a matter of increasing concern. While the toxicological effects of PET NPs have been widely researched, there is a lack of methodologies for studying their accumulation. The present study introduces a novel method to monitor the distribution of PET NPs in germinating wheat (*Triticum aestivum* L.) seeds. This involves the functionalization of superparamagnetic iron oxide nanoparticles (SPIONs) with PET NPs (PET–fSPIONs) coupled with magnetic resonance microimaging (µMRI) to provide insight into their distribution within the seed. The present study has demonstrated that PET–fSPIONs accumulate in specific regions of germinating wheat seeds, including the shoot apical meristem, the radicle, the coleoptile, the plumule, and the scutellum. Furthermore, the accumulation of PET–fSPIONs has been shown to exert a discernible effect on spin–spin relaxation (*T*_2_), as observed via MRI and quantitative *T*_2_ relaxation time analysis. The accumulation of PET NPs in embryo regions was also confirmed by SEM. Diffusion-weighted magnetic resonance imaging (DW-MRI) and non-invasive chemical shift imaging analyses demonstrated that PET NPs resulted in restricted diffusion within the highlighted areas, as well as an impact on lipid content. Our study reveals that using µMRI with fSPIONs provides a non-invasive method to monitor the biodistribution of PET nanoparticles in wheat seeds. Additionally, it offers valuable insights into the microstructural interactions of PET.

## 1. Introduction

Plastics are persistent pollutants that circulate in the primary food chain and can pose a threat to our environment. It is estimated that 390.7 million tons of plastic products are produced each year and 55 percent of global plastic waste end up in landfills [1,2]. More than 690 marine species have been affected by plastic pollution and are reported to ingest plastics, with adverse effects postulated through physical impairment, direct metabolic toxicity, or physiological effects from additives and adsorbed pollutants [3]. The major concerns regarding plastics in terrestrial systems are their observed prevalence, their transport through the soil, and the further translocation of these particles through the food chain. According to the estimations, humans are exposed to 39,000 to 211,000 micro- and nanoplastic (MPs/NPs) particles per year via drinking water bottles, air, and food [4]. Polyethylene terephthalate (PET) is one of the most widely used polymeric materials in packaging as well as textiles worldwide. PET plastic particles have been found in groundwater, drinking water, soil, sediments, and air [5,6,7,8]. The hazardous effects of PET NPs in various organisms (e.g., zebrafish, copepods) and humans have been studied [5,8,9,10]. These particles can alter cell physiology and affect cellular metabolism, leading to hepatoxicity associated with membrane dysfunction and alterations in pathways involved in energy metabolism [11]. Although there are many toxicological studies on the influence of PET NPs in various biological systems, a suitable method for tracking and internalization of these particles is still lacking.

Previous studies have attempted to track the accumulation of these particles using fluorescently labeled NPs [12,13,14]. However, the use of fluorescence techniques is challenging due to numerous factors such as background effect, scattering, and bleaching, which further affect the sensitivity and accuracy of detection. To enhance the understanding of the uptake and biodistribution of NPs, some studies focused on radiolabeled NPs [15,16,17]. For example, radiolabeled zirconium-89 (half-life, t_1/2_ ~ 3.3 days) and ^14^C-labeled (t_1/2_ ~ 5730 years) PS NPs have been used to study the biodistribution of these particles in vivo using positron emission tomography [18]. In addition, a quantitative imaging approach, radionuclide imaging tracking, has been used to follow the spatial and temporal biodistribution of various emerging contaminants, including NPs, in vivo [19]. However, it has limited applicability due to its hazardous nature and cost [20]. A previous study has focused on tracking PET NPs in vivo using inductively coupled plasma mass spectroscopy with an iridium-containing organic molecular agent [21]. Although this method provides quantitative information on the spatial and temporal distribution of NPs, it requires complex sample preparation. Furthermore, the presence of a complex organic matrix could affect the ionization efficiency of iridium, leading to potential underestimation.

Advances in magnetic resonance imaging (MRI) provide the possibility to assess the structure and metabolism of living organisms non-invasively for diagnostic and research purposes [22,23,24]. MRI has been used to study dynamic processes in plant biology using contrast agents (e.g., Gd based) [25,26]. This approach relies on the immediate impact of paramagnetic contrast agents on tissue-specific magnetic properties, predominantly altering the longitudinal relaxation times (*T*_1_). Despite the complex tissue architecture of plants, the adaptability of MRI techniques allows for effective imaging, mitigating many of the challenges associated with plant versus animal tissue differences [24]. Recently, Sarcletti et al. proposed a method for the quantification of polystyrene (PS) and polymethylmethacrylate (PMMA) NPs/MPs in water samples using iron-based contrast agents (e.g., superparamagnetic iron oxide NPs (SPIONs) with different iron cores (Fe_3_O_4_ or Fe_2_O_3_) with corresponding phosphonic acid monolayers) [25]. These contrast agents have previously been shown to be non-toxic to leukemia cells [27]. SPIONs are single-domain iron oxide nanoparticles with a core diameter between 10 nm and 100 nm [28,29]. Due to their unique distinct characteristics, SPIONs have been used in a variety of biomedical applications, including diagnostics and therapeutic applications [7,30,31]. These nanoparticles are widely used as magnetic-based *T_2_* contrast agents due to their high chemical stability, lack of toxicity, and biodegradability [11,32]. SPIONs enhance MRI imaging by causing a darkening as it influence the relaxation rates of nearby water protons, improving the contrast in the MRI images.

The unique chemical and relaxation properties of PET NPs pose significant challenges to their detection via traditional MRI methods, underscoring the need for innovative approaches. The use of MRI contrast agents attached to PET NPs can be a feasible approach to track the localization of these NPs in biological organisms, as they can influence the relaxation of the nearby protons. Attachment of another type of NPs, namely PS to functionalized SPIONs, has been shown in a previous study for the determination of NPs in water samples [33].

In this proof-of-concept study, we attached PET NPs to functionalized SPIONs (PET–fSPIONs) and presented a non-invasive method to monitor the accumulation of PET NPs in germinating wheat (*Triticum aestivum* L.) seeds using µMRI. Wheat is a widely grown crop, accounting for 30% of global crop consumption. Given the widespread presence of PET NPs, comprehending their accumulation and effects on metabolism is essential [34]. Our results show that PET NPs bound with fSPIONs can be non-invasively monitored using µMRI. Furthermore, we show that PET NPs significantly influence the microstructural changes in germinating wheat seeds. Our findings demonstrate the potential of µMRI in advancing our understanding of nanoparticle interactions and provide valuable insight for future research in this field.

## 2. Results and Discussion

In this study, we aimed to develop a biocompatible method using fSPIONs as a contrast agent to track PET NPs in wheat seeds non-invasively using µMRI. The PET NPs and fSPIONs were synthesized, and their successful agglomeration was verified through comprehensive characterization. We assessed the relaxivity of PET–fSPIONs to ensure their effectiveness as MRI contrast agents. µMRI was then employed to visualize the distribution of PET–fSPION in germinating wheat seeds, providing detailed insights into their localization and interaction.

### 2.1. Characterization and Analysis of PET–fSPIONs

At first, the stable PET NPs and fSPIONs were prepared as described previously [35,36]. Subsequently, PET NPs were attached with fSPIONs to generate PET–fSPIONs. To assess the interaction between PET NPs and fSPIONs, characterization was performed using transmission electron microscopy (TEM), dynamic light scattering (DLS), and Fourier transform infrared spectroscopy (FTIR).

**Figure 1:**
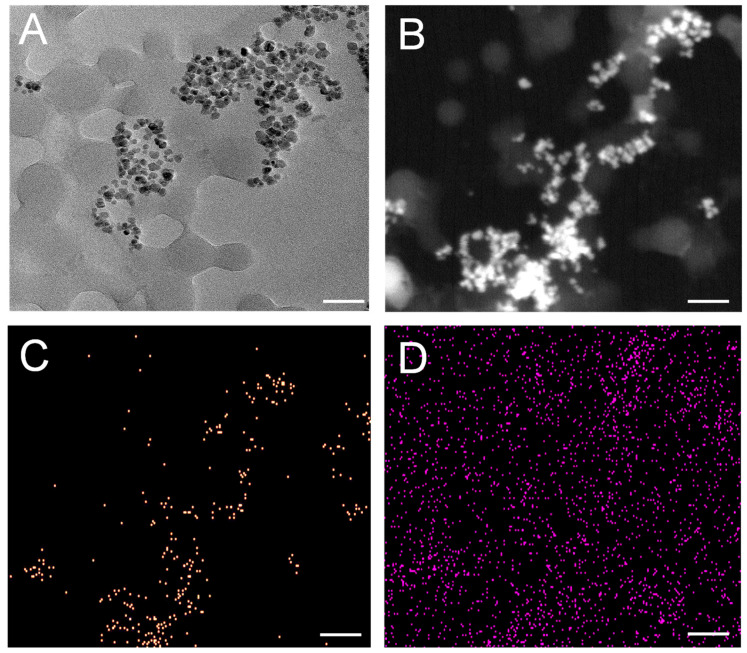
TEM, STEM, and EDX characterization of PET–fSPIONs. (**A**) TEM images of PET–fSPIONs. (**B**) HAADF STEM image of PET–fSPIONs showing that fSPIONs are well dispersed around PET NPs spheres. (**C**,**D**) STEM-EDX map of PET–fSPIONs where net intensity EDX signals of iron in yellow (**C**) and of carbon in purple (**D**) is shown. Scale bars: 50 nm.

Detailed insights into the morphology of both PET NPs and PET–fSPIONs assessed via TEM are shown in Figure 1. TEM imaging revealed that the PET NPs were spherical and there was an interaction between fSPIONs within the PET NPs (Figure 1A). This interaction is observed as the fSPIONs consistently agglomerate around PET NPs spheres. The PET NPs have an average size of ~44 nm, while the fSPION particles have an average size of 8.8 nm. To investigate details of the distribution of fSPIONs around PET NPs, material contrast imaging was performed by high-angular dark-field scanning transmission electron microscopy (HAADF-STEM) and energy-dispersive X-ray spectroscopy (EDX). The HAADF-STEM imaging shows an overview of fSPIONs around PET NPs (Figure 1B). The STEM-EDX map illustrates the elemental distribution with net intensity EDX signals. The yellow signals correspond to iron, indicating the presence and distribution of the fSPIONs within the PET NPs (Figure 1C). Additionally, carbon signals, depicted in purple, emphasized the carbon-rich nature of the PET NPs as well as the carbon support film of the TEM grid (Figure 1D). The size distribution of PET NPs and fSPIONs is shown in Supplementary Figure S1.

**Figure 2:**
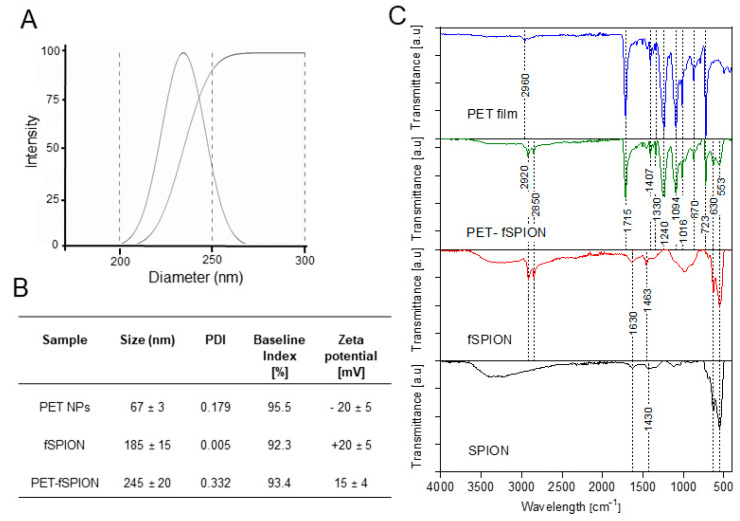
Characterization of PET NPs with and without functionalized fSPIONs. (**A**) Hydrodynamic diameter distribution of PET–fSPIONs. (**B**) Particle size, PDI, and zeta potential of PET, fSPIONs, and PET–fSPIONs determined by DLS. (**C**) ATR-FTIR spectra of PET film, PET–fSPIONs, fSPIONs, and SPIONs.

DLS analysis was employed to assess the hydrodynamic size distribution and zeta potential of PET NPs, fSPIONs, and PET–fSPIONs, as shown in Figure 2A,B. This analysis revealed that the hydrodynamic sizes of PET NPs, fSPIONs, and PET–fSPIONs were 67 ± 3 nm, 185 ± 15 nm, and 245 ± 20 nm, respectively (Figure 2B).

The difference in particle sizes observed by TEM and DLS may be due to the distinct principles underlying these techniques. TEM provides high-resolution images of the dry core size of individual particles under vacuum conditions, excluding the influence of the surrounding medium. In contrast, DLS measures the hydrodynamic diameter of particles suspended in solution, which includes not only the core size but also the surrounding solvent layer and any surface coatings or functionalization. Additionally, DLS is sensitive to particle agglomeration and interactions in solution, which can further increase the observed size. These factors may have contributed to the significantly larger sizes observed by DLS compared to TEM in our study.

Zeta potential measurements demonstrated that PET NPs exhibit a negative charge (−20 ± 5 mV), whereas fSPIONs displayed a positive charge (+20 ± 5 mV). The combination of PET NPs with fSPIONs in PET–fSPIONs yielded a composite with a surface charge of +15 ± 4 mV, nearly as positive as that of pure fSPIONs. The relatively larger size of the PET–fSPION composite compared to the individual components could be due to the formation of a stable and integrated nanostructure, potentially due to strong electrostatic interactions and physical entanglement between the particles. Furthermore, the hydrodynamic size of PET–fSPIONs (245 ± 20 nm) indicates that these particles are within an effective range for plant tissue penetration [34]. The positive zeta potential of PET–fSPIONs suggests that the fSPIONs’ surface properties predominantly influence the overall charge of the composite. The reduced absolute value of the zeta potential in the composite compared to pure fSPIONs (+15 mV vs. +20 mV) might be due to the partial neutralization of charges, indicating electrostatic interaction between the components. The polydispersity index (PDI) values suggest that both the PET–NPs and PET–fSPIONs are homogeneous in solution, show low tendencies towards aggregation, and remain stable throughout the measurements. For detailed characteristics of each particle group, refer to Figure 2B.

FTIR spectrum of amorphous PET film, PET–fSPION, fSPION, and SPION is shown in Figure 2C. The FTIR analysis revealed specific vibrational modes associated with the chemical bonds present in the samples. The PET film exhibited strong stretching vibrations of the C–O bond *ν*(C–O) at 1715 cm^−1^ and vibrations associated with *ν*(C–O) (esters and ethers) around 1240 cm^−1^. Weak absorption bands corresponding to alkyl *ν*(C–H) and aryl groups, as well as characteristic bending vibrations of aromatic compounds, were observed at 2960 cm^−1^ and 1407 cm^−1^, respectively.

These findings are consistent with the molecular structure of PET [35]. For both fSPIONs and commercially purchased SPIONs (γ-Fe_2_O_3_), the stretching bands appeared in the low wave number range of 500**–**750 cm^−1^. Additional weak signals were detected at 1430 cm^−1^ (associated with CH_n_ vibrations), 1630 cm^−1^ (related to C-C vibrations), and around 3300 cm^−1^ (typical of *ν*(O-H) vibrations). The fSPIONs exhibited prominent signals corresponding to alkyl chains at 2920 cm^−1^ *ν*(C-H) and 2850 cm^−1^ *ν*(N-C), as expected based on the structural characteristics [36]. A broader peak around 1000 cm^−1^, attributed to the phosphate group, was also observed. The absorption bands characteristic of SPIONs were still present in fSPIONs but exhibited different transmittance properties. When mixing PET NPs with fSPIONs and analyzing PET–fSPIONs, the same characteristic bands of PET, as well as the fSPIONs, were observed. These results suggest that coupling of PET NPs with fSPIONs in PET–fSPIONs did not alter the chemical properties of PET, as well as fSPIONs, and it remained intact during the mixing process. Thus, FTIR analysis indicates that the attachment of PET NPs with fSPIONs was successful. The adsorption of fSPIONs to PET NPs may have been facilitated by both electrostatic and van der Waals interactions, as evidenced in previous studies where similar processes have been observed in the use of other types of NPs, including PS and PMMA [33].

### 2.2. Relaxivity of PET–fSPIONs and fSPIONs

**Figure 3:**
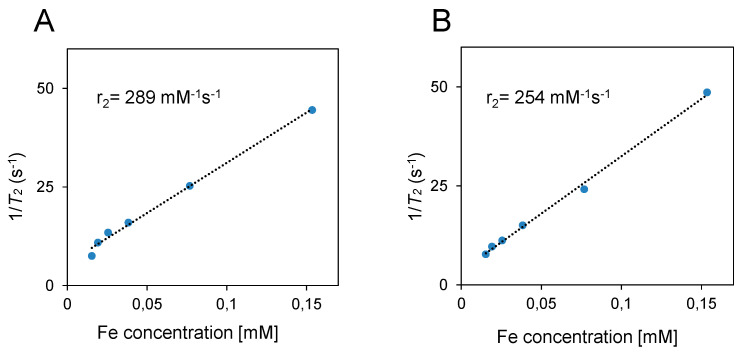
The relaxivity of fSPION (**A**) and PET–fSPION (**B**) was determined at different concentrations using µMRI. The relaxation rates 1/*T*_2_ were plotted against the Fe concentration. The relaxivities (r_2_) were calculated from the linear regression slopes. The r_2_ for fSPION was 289 mM^−1^s^−1^ with an R^2^ of 0.9933, while the r_2_ for PET–fSPION was 254 mM^−1^s^−1^ with an R^2^ of 0.9970.

To investigate whether the attachment of PET NPs with fSPIONs can influence their relaxation properties, we analyzed the *T*_2_ relaxivity of fSPIONs and PET–fSPIONs. The standard curve using various concentrations of fSPIONs and PET–fSPIONs was made to obtain a transverse relaxation rate (R^2^) value of 0.9933 for transverse relaxivity (r_2_) SPIONs and an R^2^ value of 0.9970 for r_2_ PET–fSPIONs (Figure 3A**,**B). R^2^ values show the fit quality of the data to the regression model. The standard curve equation was as follows: y = 254.63x + 5.6377 (PET–fSPION) and y = 289.4480x + 3.5588 (fSPION), where x represents the concentration of Fe^2+^ ions (mM) and y is the relaxation rate (s**^−^**^1^). The results indicate a slight variation in r_2_ values between fSPION (289 mM**^−^**^1^s**^−^**^1^) and PET–fSPION (254 mM**^−^**^1^s**^−^**^1^), respectively. The plotted transverse relaxation rate, R^2^ (1/*T*_2_), exhibited a linear correlation with fSPION and PET–fSPION concentration (mM). The r_2_ value of our fSPION showed a relaxation property-altering effective *T*_2_ value with an r_2_ of 289 mM**^−^**^1^s**^−^**^1^. This finding is consistent with previous measurements of SPIONs integrated with a chitosan-based polymer, which exhibited an r_2_ value of 294.8 mM**^−^**^1^s**^−^**^1^ at the same Fe^2+^ ion concentration used in our study. This consistency extends across various cluster sizes in other studies with 150 nm clusters of 8 nm particles, 100 nm clusters of 7 nm particles, and 50 nm clusters of 8 nm particles exhibiting 294 mM**^−^**^1^s**^−^**^1^, 294 mM**^−^**^1^s**^−^**^1^, and 227 mM**^−^**^1^s**^−^**^1^ in relaxivity, respectively [37,38,39]. The slight variation in magnetic relaxivity between PET–fSPION and fSPION might be related to the changes in the local magnetic field within the solution resulting from the introduction of non-magnetic PET NPs. Thus, even non-magnetic materials like plastics can affect the local magnetic field to some extent due to their physical presence [40]. This is a phenomenon known as the magnetic susceptibility of materials [41]. Although the effect of PET NPs on the magnetic field is minimal, it can still alter the local magnetic environment, resulting in slightly different relaxivity values for PET–fSPION compared to fSPION. This has been explained in earlier studies concerning other non-magnetic materials [41].

### 2.3. Localization of PET NPs in Wheat: Insights from SEM

**Figure 4:**
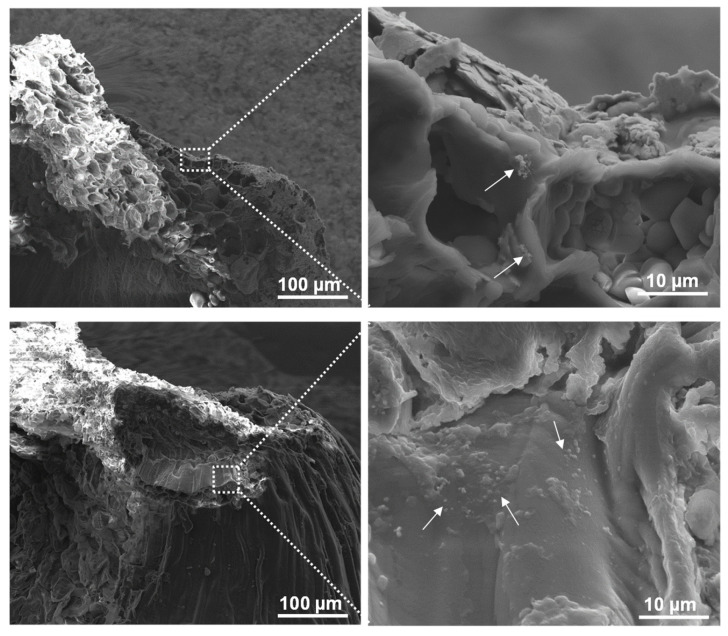
SEM images showing the localization of PET–fSPIONs in wheat seeds. Seeds were treated with a PET–fSPION. Aggregations of PET–fSPIONs are visible (white arrows). The area in the white squares is magnified to the right, highlighting the detailed interaction between the nanoparticles and the embryo surface.

Scanning electron microscopy (SEM) was employed to track the distribution and morphology of the PET NPs and PET–fSPION particles within wheat seeds (Figure 4 and Figure S2). The SEM analysis revealed the presence of PET NPs as aggregates, exhibiting a distinct chain-like formation. This morphological evidence indicates the translocation of PET NPs and their specific localization within the embryo region of the wheat seed. Moreover, these particles were also seen in the endosperm and crease regions of wheat seed (Figure S3). These results suggest that PET–fSPIONs were absorbed by seeds during imbibition and effectively penetrated through the seed coats and entered seed tissues. These results are in line with earlier studies, where other types of nanoparticles have been shown to be absorbed by the seeds during imbibition [42]. Direct absorption of nanoparticles in seeds is known to occur by entering the coat via parenchymatic intercellular spaces, accompanied by diffusion in the cotyledon [43].

### 2.4. Impact of PET–fSPIONs on Spin–Spin Relaxation in Wheat Seeds

**Figure 5:**
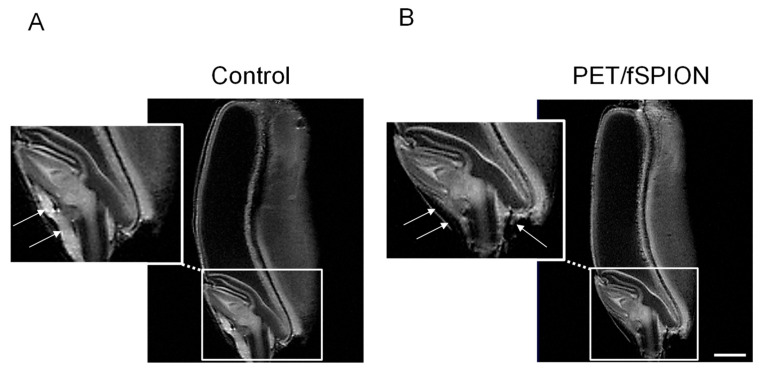
Representative MRI images of wheat seeds: (**A**) shows the control group with no treatment (e.g., DI water), while (**B**) shows seeds treated with PET–fSPIONs. Arrows indicate the presence of PET–fSPIONs. Insets below each image provide magnified views of the regions highlighted by the dashed boxes, illustrating the internal structure of the wheat seeds. Measurements performed using a RARE sequence with the following parameters: TR = 2000 ms, TE = 9.15 ms, slice thickness = 0.3 mm, RARE factor = 4, FOV = 8 × 8 cm, number of scans = 256, in–plane spatial resolution = 0.31 × 0.31 mm^2^, total acquisition time ≈ 9h 6 min. Scale bar: 1 mm.

Representative *T*_2_-weighted MRI images of germinating wheat seed treated with only water (control) or PET–fSPIONs are shown in Figure 5. The embryo region is shown in magnification. As PET–fSPIONs are magnetic and have a stronger effect on the local magnetic field, they shorten the *T*_2_ relaxation times of neighboring protons [42]. Thus, the presence of PET–fSPIONs at various locations in the seed is clearly visible as darker areas in the images (indicated by arrows in the magnified area). A marked reduction in signal intensity was also observed in the region around the root cap and root sheath.

**Figure 6:**
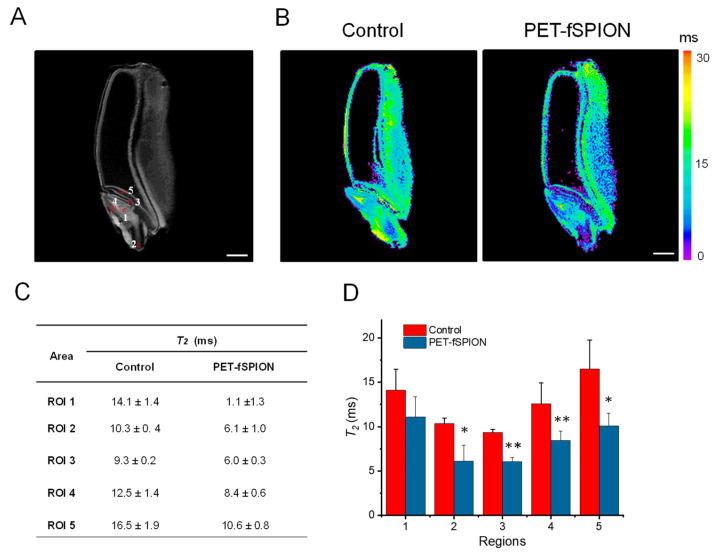
*T*_2_-weighted MRI analysis of control and PET–fSPION-treated wheat seeds. (**A**) *T*_2_-weighted MRI image of wheat seed, showing various regions used for *T*_2_ measurements. (**B**) Representative color-coded *T*_2_ maps of control and PET–fSPION-treated wheat seed. The color scale indicates *T*_2_ relaxation times ranging from 0 to 30 ms. Short *T*_2_ values are observed in the PET–fSPION-treated wheat in comparison to the control. (**C**) Quantitative *T*_2_ relaxation time values in different ROIs. Data are presented as mean ± standard deviation for each ROI. (**D**) Reliability test of *T*_2_ measurements was performed using ANOVA to accomplish pairwise comparisons of the data. At the 0.05 level, the *T*_2_ population means of control and PET–fSPION-exposed wheat are significantly different (* *p* < 0.05, ** *p* < 0.01). Data represent the mean *T*_2_ in ms ± SD with error bars. *T*_2_ measurements performed using a 2D MSME sequence with the following parameters: TR = 2000 ms; sixteen TEs ranging from 4.3 ms to 68.8 ms; slice thickness = 0.3 mm; FOV = 8 × 8 cm; in-plane spatial resolution = 0.31 × 0.31 mm^2^; acquisition time = 2 h 16 min. Scale bar: 1 mm.

These observations are further supported by quantitative *T*_2_ relaxation analysis (Figure 6). The shortening of *T*_2_ due to penetration of PET–fSPIONs was observed in different areas of the embryos, including the shoot apical meristem (see region of interest 1, ROI 1), radicle (ROI 2), coleoptile (ROI 3), plumule (ROI 4), and scutellum (ROI 5), as illustrated in Figure 6A. A colour *T*_2_ map, as shown in Figure 6B, also clearly indicates the reduction in *T_2_* due to the penetration of PET–fSPIONs in various locations within the embryo. The *T_2_* relaxation time values of all five selected ROIs in the embryo were quantitatively analyzed (Figure 6C). Figure 6D represents the results of the one–way ANOVA statistical analysis performed on the *T*_2_ values from selected ROIs. The results indicated that there were significant changes in the *T*_2_ values in the radicle and scutellum (*p* < 0.05), with more significant changes in the coleoptile and plumule regions (*p* < 0.01) when exposed to PET–fSPIONs compared to the control. In contrast, shoot apical meristem exhibited a less pronounced effect on *T*_2_ relaxation time.

These results indicate that regions such as the coleoptile and plumule, which play an active role in early seedling development, showed higher accumulation of PET–fSPIONs. The coleoptile is a protective sheath covering the young shoot of the embryo that helps guide the shoot through the soil, and the plumule is the part of the embryo that develops into the shoot system, including the first true leaves of the plant. These areas are highly metabolically active. The enhanced permeability and retention effect of other nanoparticles in these regions with high metabolic activity and permeability has been documented [44,45].

### 2.5. Diffusion MRI Measurements

The regions that show a high accumulation of PET NPs were further analyzed by diffusion-weighted MRI (DW-MRI), a technique that enables the visualization of both the diffusion restriction and microstructural alterations resulting from PET NPs accumulation.

**Figure 7:**
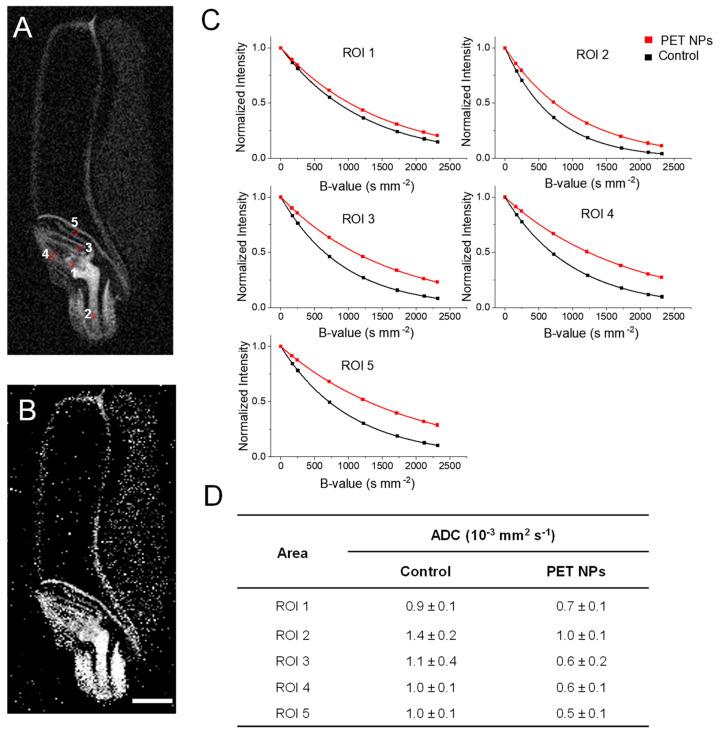
Diffusion-weighted MRI in embryo regions of control and PET NP–treated wheat seed. (**A**) A diffusion image of control wheat showing regions of interest (ROI) located in various embryo regions of wheat. The ROIs are located in the shoot apical meristem (ROI 1), in the radicle of wheat (ROI 2), in coleoptile (ROI 3), in plumule (ROI 4), and scutellum (ROI 5). (**B**) Representative ADC map image of control wheat generated through Bruker internal ‘dtraceb’ algorithm. It shows the distribution of ADC values where higher ADC appears bright and lower ADC appears darker. (**C**) Signal decay curve in respective ROIs as shown in (**A**). (**D**) Table of calculated ADC values from reinterest is shown in (**A**). Diffusion measurements were performed using a spin-echo pulse sequence containing a pair of mono–polar diffusion–sensitizing gradients (TR = 2000 ms; TE = 14 ms; diffusion gradient duration = 2 ms and gradient separation = 8 s; effective B-values range: 100, 500, 1000, 1500, 2000, 2200 s/mm^2^). Scale bar: 1 mm.

The apparent diffusion coefficient (ADC) is a well-established marker of cellular integrity and tissue structure [46]. It was used to assess the intracellular diffusion barrier. DW-MRI measurements were performed in selected ROIs which show PET–fSPION accumulation. Figure 7A shows the diffusion image of wheat seed and indicates selected ROIs. The ADC map generated through the ‘dtraceb’ algorithm illustrated the diffusion behavior across the seed (Figure 7B). A normalized mono-exponential fitting curve for each selected ROI, in embryo regions, is shown in Figure 7C. In all ROIs, PET NP-treated seeds show attenuated signal decay, indicating restricted diffusion compared to control wheat. ADC values were significantly lower in PET NP-treated seeds across all ROIs (Figure 7D). The coleoptile (ROI 3), plumule (ROI 4), and scutellum (ROI 5) show a significant reduction in ADC values. These results indicate that the accumulation of PET NPs in these areas causes diffusion restriction, either directly or due to their interaction with the microstructure of the wheat seed embryo.

### 2.6. Chemical Shift Imaging

**Figure 8:**
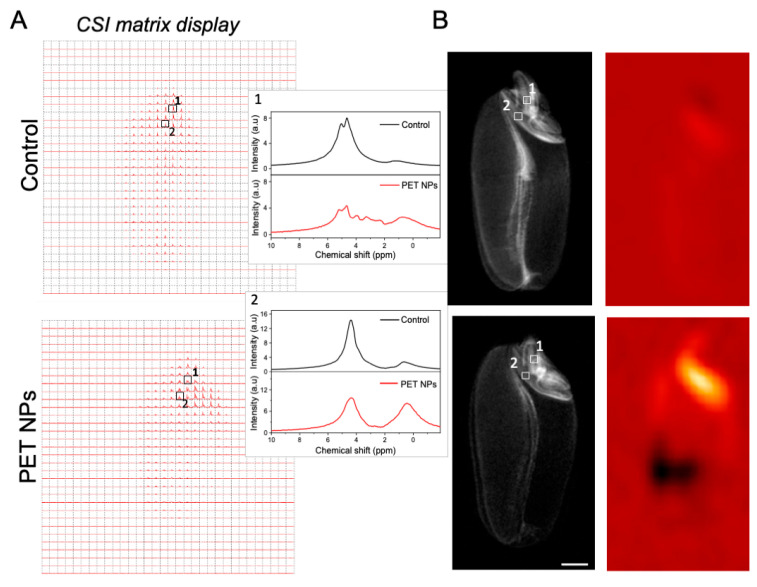
Chemical shift imaging. (**A**) Matrix display of chemical shift imaging spectra of control and PET NP-treated wheat seeds. Spectra from radicle (1) and scutellum (2) regions are shown in magnification. Signals between ~0.5 to 3 ppm are from lipids and 4.7 ppm from water. Overlapping signals from carbohydrates (glucose, fructose, or other sugars) between ~3 to 5 ppm are present in radicle region in PET NP-treated samples. (**B**) Corresponding CSI voxel intensity thresholding of lipid signals which was reconstructed as a color map and overlaid with corresponding *T*_2_-weighted RARE images using the Bruker CSI Visualization Tool. CSI data were recorded with a TR = 2000 ms; TE = 15 ms and slice thickness was 3 mm. Total averages were 256. Resolution obtained was 0.625 × 0.625 mm^2^. Spectral width used was 10 kHz (13.33 ppm) and the 16 × 16 matrix was reconstructed into 32 × 32 voxels. Scale bar: 1 mm.

Non-invasive chemical shift imaging (CSI) was used to analyze the content and distribution of metabolites in wheat seed at the spectroscopic level. CSI is particularly adept at mapping the spatial distribution of hydrogen nuclei, which are typically associated with either water or lipid molecules (Figure 8). In Figure 8A, the CSI data from scutellum and radicle regions are presented as a matrix array of spectra. The peak between ~0.5 and 3 ppm corresponds to the aliphatic protons -(CH_2_)n- of carbon atoms (Figure 8A). This suggests that this region contains lipids that might have been mobilized from stored triacylglycerol during the early stages of seed germination [47,48,49]. The peak at 4.7 ppm is from the water signal.

Wheat seeds treated with PET NPs showed an increase in lipid content and a decrease in water content in the scutellum as well as in radicle regions during the early stages of germination (Figure 8). While some lipid content is necessary for seed viability, excessively high levels might impede other critical functions. For example, high lipid accumulation may affect water uptake or oxygen diffusion, which could have a negative impact on germination rates and overall seedling vigor [50,51,52]. The observed increase in lipid content may be indicative of a stress response to PET NPs exposure, as has been previously documented in plant studies with other NPs [53]. The underlying mechanisms by which exposure to PET NPs leads to enhanced lipid accumulation remain unclear and necessitate further investigation. To visualize the spatial distribution of lipids, a color map is reconstructed from the lipid signal and displayed and overlaid on an MR image of the same slice (Figure 8B).

The second noticeable peak in the radicle region, occurring in the range of ~3 to 5 ppm, is characteristic of protons found in overlapping carbohydrate molecules [54,55,56]. These are likely present due to the degradation of starches and other polysaccharides as the seed activates its metabolism. To contextualize these findings, we refer to the literature on the effects of other nanoplastics, namely PS NPs on starch granules during wheat seed germination [57]. It has been observed that PS NPs can induce higher α-amylase activity, leading to significant degradation of starch granules [57]. α-amylase typically initiates the hydrolysis of starch by pitting the surface of the starch granule and then penetrating into the interior, hydrolyzing the granule from the inside out. They observed that starch granules have a rougher and eroded appearance in PS NP-treated seeds, implying increased α-amylase penetration and higher starch decomposition [57]. By analogy, we hypothesize that, in our study, PET NPs may have similar effects leading to high degradation of starch and accumulation of sugars as seen in our CSI data (Figure 8). Despite the elevated level of sugars observed in the radicle of PET NP-treated seeds, it was not utilized for radicle growth. Rather, it appears that these sugars may have been accumulated as a means of coping with the stress generated by PET NPs treatment.

## 3. Materials and Methods

### 3.1. Chemicals

All chemicals were purchased from Sigma-Aldrich (St. Louis, MO, USA) unless otherwise stated. 12-dodecylphosphonic acid-N, N-dimethyl-N-octadecylammonium bromide (PAC12NC18) was purchased from Sikemia, France. SPIONs (used as a hypernym for Fe_2_O_3_) with a size below 45 nm were provided by COMAR Chemicals, South Africa.

### 3.2. Preparation of PET–fSPIONs

Initially, PET NPs were prepared according to the previous method with slight modifications [58,59]. Briefly, 100 mg of amorphous PET (Goodfellow, Friedberg, Germany) was dissolved in 10 mL of hexafluoroisopropanol (1% *w*/*v*) at room temperature for 12 h. The PET solution was then transferred to the buret and the solution was added dropwise to ice-cooled deionized (DI) water (200 mL) while stirring continuously. To eliminate larger particles, the suspension was filtered (type 12, cellulose membrane, 125 mm diameter, Roth, Germany). The organic solvent was then removed from the solution using a rotary evaporator (Heidolph Instruments, Wood Dale, IL, USA) at elevated temperature and reduced pressure (50 °C, ~250 mbar). The NPs were then allowed to settle in the cylinder for 2 h and the upper 40 mL suspension was collected. The concentration of NPs was determined gravimetrically by drying 2 mL of the suspension in a pre-dried polymer pellet (3×) at 50 °C for 24 h and then weighing to quantify the residue. The stock concentration of PET NPs used was 0.35 mg/mL. The prepared solution was stored at room temperature, and it was stable for several weeks without any sedimentation.

To attach PET NPs to SPIONs, the SPIONs were first functionalized. Functionalized SPIONs (fSPIONs) were prepared using gamma-Fe_2_O_3_ core labeled with PAC_12_NC_18_ according to the following established procedure [33,60,61]. Briefly, PAC_12_NC_18_ (20 mM), as well as SPIONs (3 mg/mL), were dispersed in methanol. PAC_12_NC_18_ and SPIONs were mixed in a ratio of 3:10 and sonicated in a SONOCOOL 255 (Bandelin, Berlin, Germany) for 30 min. The dispersion was then centrifuged to collect fSPIONs. The collected SPIONs were washed twice in methanol and the supernatant of the second centrifugation step was transferred into DI water using a Hei-Vap Advantage rotary evaporator (Heidolph Instruments, Germany) at 60 °C and 337 mbar. The final fSPION stock concentration was 0.35 mg/mL.

Subsequently, PET NPs were mixed with fSPIONs in a ratio of 2:1 (*v*/*v*) to prepare PET–fSPIONs.

### 3.3. Dynamic Light Scattering (DLS)

DLS (ZetaPALS, Brookhaven, NY, USA) was used to determine the size of the fSPIONs, PET NPs, and PET–fSPIONs in water. For analysis of PET–fSPION, the solution was first diluted tenfold with water before the DLS measurement. Samples were allowed to equilibrate in the sample compartment at 25 °C for at least 2 min and measured in acrylic cuvettes (10 × 10 × 45 mm, Sarstedt, Germany) at 25 °C. Each measurement was carried out three times and zeta potential measurement was conducted in 10 successive runs. The hydrodynamic diameter and zeta potential (according to the Smoluchowski equation) of PET NPs were evaluated [58,62].

### 3.4. Attenuated Total Reflectance Fourier Transform Infrared Spectroscopy (ATR-FTIR)

ATR-FTIR was performed on a Bruker Alpha II FTIR spectrometer (Bruker Optik GmbH, Ettingen, Germany) equipped with a Diamond Crystal ATR attachment. The spectrum of the diamond crystal was obtained as background. Analyzed samples included fSPION, SPION, PET–fSPION, and PET film. For analysis, 20 µL of each sample (fSPION and PET–fSPION) solution was applied to the ATR crystal and then dried at 60 °C for 15 min. Solid samples, such as SPION and PET film, were directly placed on the ATR crystal absorbance spectra and were recorded between 4000 and 399 cm**^−^**^1^ with a resolution of 2 cm**^−^**^1^ and 128 averaged scans. Data acquisition was performed using Opus 7.8 software (Bruker Optik GmbH, Germany) and the acquired data were further plotted using OriginPro v.8 (OriginLab, Northampton, MA, USA).

### 3.5. Scanning Electron Microscopy (SEM)

Wheat seeds were soaked in DI water or PET–fSPION suspension for 24 h. Subsequently, the samples were fixed with 4% formaldehyde (FA) for 24 h and rinsed with PBS buffer. After fixation, the samples were manually cut in half with a scalpel and dried at 50 °C for 6 h. Subsequently, they were mounted onto sample holders using silver DAG (PLANO), and platinum coating, approximately 5 nm thick, was applied using a BAL-TEC SCD 050 sputter coater for 40 s at 40 mA, while maintaining a working distance of 50 mm. SEM images were then taken using a NOVA NANO**^®^** SEM 200 (Thermo Fisher, Dreieich, Germany) equipped with a Schott field emitter. The imaging conditions were set to a high voltage of 10 kV with magnifications of 700× and 10,000×, and a working distance of 5.5 mm for each image.

### 3.6. Transmission Electron Microscopy (TEM)

TEM was employed for the characterization of PET–fSPIONs. The sample preparation was performed by transferring a droplet (10 μL) of PET–fSPION suspension onto a holey carbon TEM-grid, which was allowed to settle and dry at room temperature. TEM analysis was performed using a JEM2100Plus microscope (Jeol, Tokyo, Japan). The microscope is equipped with a LaB6 filament and high-resolution pole piece and was operated at an accelerating voltage of 200 keV. Elemental analysis was performed by energy-dispersive X-ray (EDX) analysis using a windowless Optima T–30 detector (EDAX, UK). Furthermore, images were acquired with a 4K ultrafast CMOS camera system (TVIPS, Germany) and analyzed with the associated EMMeasure software, Version 4.09.53 (TVIPS, Bayern, Germany). High-angle annular darkfield (HAADF) scanning transmission electron microscopy (STEM) was employed to obtain material contrast imaging of the heavy Fe_2_O_3_ and the lighter PET NPs. The acquisition of HAADF STEM images was performed by the TEM center software (Jeol, Japan), and evaluation of the images and size of the particles was evaluated by SightX-Viewer (Jeol, Japan).

### 3.7. Determination of Iron Content in fSPIONs

Prior to evaluating the relaxivities of fSPIONs and PET–fSPIONs, the iron concentration was determined using the 1, 10-phenanthroline method [63]. Briefly, a standard solution of iron was prepared by dissolving iron ammonium sulfate hexahydrate in water to achieve a concentration of 70 mg/L (equivalent to 0.178 mmol/L), with a volume of 200 mL. To this, 2.5 mL of concentrated sulfuric acid was added before diluting to 1 L with water in a volumetric flask. Calibration standards were created at concentrations of 0, 0.7, 3.5, 7, 14, and 24.5 mg/L from the iron standard, with DI water serving as the blank. The appropriate volumes, were transferred into volumetric flasks. To each flask, including the blank, the following were added: 1 mL of a 100 g/L hydroxylamine solution, 10 mL of a 1 g/L 1, 10-phenanthroline solution, and 8 mL of a 98.4 g/L sodium acetate solution. The fSPION sample was prepared in the same manner as the calibration standards, with an iron concentration of approximately 7 mg/L. After allowing all solutions to stand for 10 min for development, the absorbance was measured thrice at 510 nm using a Shimadzu 1900i UV/VIS (Columbia, Portland, OR, USA) spectrometer equipped with a TCC240A temperature control system. The iron concentration in the fSPION sample was then calculated from the calibration curve established by the absorbance readings of the standard solutions.

### 3.8. Relaxivity Measurement of fSPIONs and PET–fSPIONs

The performance of fSPIONs as a contrast agent was assessed by evaluating the relaxivities of fSPIONs and PET–fSPIONs using a 200 MHz (4.7 T) MRI system. Samples were linearly diluted either in DI water or PET NPs. *T*_2_ values were recorded using a spin-echo pulse sequence. The imaging parameters for the experiments were the following: number of averages (NA) = 64, number of slices = 3 with a slice thickness of 0.5 mm, number of echoes = 40 with echo spacing = 4.28, repetition time (RT) = 2 s, effective spectral bandwidth = 100 kHz, filed of view (FOV) = 20 × 20 cm^2^, matrix size = 128 × 128, providing an effective in-plane resolution of 0.078 × 0.078 mm^2^, and a voxel resolution of 10 × 10 × 3 mm^3^. The total acquisition time for the experiment was 4 min.

### 3.9. MRI Experiments

The MRI experiments were conducted using a Bruker 300 MHz (7T) or 200 MHz (4.7T) vertical bore system (Bruker Biospin GmbH, Germany). A birdcage transmit/receive radiofrequency (RF) coil with an internal diameter of 10 mm or 5 mm was utilized at magnets. The workstation was connected to a Linux operating system running Paravision 5.1 (at 7T) or Paravision 360 v2.1 (at 4.7T) imaging software (Bruker Biospin GmbH, Germany). All measurements were conducted at room temperature.

Prior to imaging, wheat samples were imbibed for 24 h in DI water (control) and PET NPs with and without fSPIONs in a round 96-well microplate (Greiner Bio-One, Frickenhausen, Germany). For microimaging, the imbibed seeds were carefully surface-dried with a paper towel and inserted into an NMR tube and the ends of the glass cylinder were sealed with parafilm (American Can Comp., Greenwich, CT, USA) to avoid possible drying during the MRI experiment. The seeds were imaged with multi-slice multi-echo (MSME), rapid acquisition with relaxation enhancement sequences (RARE), diffusion-weighted imaging (DWI), and chemical shift imaging (CSI).

Each measurement started with a gradienT–echo sequence to determine the position and to select the desired region for further experiments. For anatomical imaging, the RARE sequence was used. RARE, a rapid imaging sequence, utilizes an RF excitation pulse succeeded by a series of refocusing pulses [64]. This technique generates multiple RF spin echoes, enabling faster image acquisition by capturing more than one k-space line during each repetition. The imaging parameters for the RARE sequence were as follows: echo time = 20 ms, RT = 2 s, NA = 64, rare factor = 4, number of slices = 10 with a slice thickness of 0.2 mm, FOV = 10 × 10 cm^2^, effective in-plane resolution of 0.078 × 0.078 mm^2^, and a voxel resolution of 10 × 10 × 3 mm^3^.

*T*_2_ values were measured using the MSME sequence, based on the Carr–Purcell–Meiboom–Gill (CPMG) sequence. In this method, transverse magnetization from a 90-degree pulse is refocused by a train of 180-degree pulses, generating a series of echoes [65,66]. The imaging parameters for in vivo experiments were the following: NA = 64, number of slices = 3 with a slice thickness of 0.5 mm, number of echoes = 16 with echo spacing = 4.28, TR = 2 s, effective spectral bandwidth = 100 kHz, FOV = 8 × 8 cm^2^, matrix size = 256 × 256, providing an effective in-plane resolution of 0.078 × 0.078 mm^2^ and a voxel resolution of 10 × 10 × 3 mm^3^. The total acquisition time for the experiment was 2 h and 16 min.

Diffusion measurements were performed using a spin-echo pulse sequence with a pair of mono-polar diffusion-sensitizing gradients [67]. Gradient orientations were evenly distributed in one direction. The effective B-values (diffusion weighting value) range was as follows: 100, 500, 1000, 1500, 2000, and 2200 s/mm^2^. A diffusion gradient duration (δ) of 2 ms was utilized in conjunction with an 8 ms diffusion gradient separation (Δ), resulting in a total TR and TE of 2000 and 14 ms, respectively. In order to achieve a sufficient signal-to-noise ratio, 12 averages were acquired, resulting in an overall acquisition time of 12 h. The FOV was 8 × 8 mm^2^, matrix size 256 × 256, and the slice thickness was set to 0.3 mm, resulting in a spatial resolution of 0.031 × 0.031 mm^2^.

Multi-voxel spectroscopic information was acquired using CSI in the spin-echo slab-selective mode. CSI employs two orthogonal phase encoding steps with pulsed gradients, capturing a pure spectroscopic echo during acquisition, distinct from the conventional readout gradient used in imaging. The simultaneous achievement of spatial and spectral resolution allows for the generation of multi-voxel spectra, resulting in spatial distribution maps of individual metabolites [68,69]. To improve the spatial response function, a Hanning function-weighted k-space acquisition scheme was employed, following the Bruker ‘weighted’ measurement method. The fundamental measurement parameters included TE = 15 ms, TR = 2000 ms, matrix size = 16 × 16, FOV = 10 × 10 mm^2^, slice thickness = 3 mm, resolution = 0.625 × 0.625 mm^2^, and the number of scans = 4096. Data underwent reconstruction into a 32 × 32 matrix size with linear smoothing for display purposes. Excitation and refocusing utilized Sinc3 pulses with a bandwidth of 8012 Hz. Echoes were acquired in 2048 points over 204.80 ms, yielding a spectral resolution of 2.4 Hz per point, and a spectral width of 10 kHz (13.3 ppm). Magnetic field homogeneity in the selected volume was optimized through water resonance shimming. VAPOR suppression, with a duration of 625 ms, efficiently saturated the water signal. Interpulse RF delays between seven hermite-shaped CSI modules were set at 150, 80, 160, 80, 100, 37.2, and 15 ms. The RF bandwidth was 900 Hz, and the excitation offset was −75 Hz (−0.1 ppm).

### 3.10. Data Processing

To calculate *T*_2_, ROIs were drawn on images using an image sequence analysis package (Paravision 2.1, Bruker) using a fit function [y = A + C × exp (−t/*T_2_*)], where A = absolute bias, C = signal intensity, and *T*_2_ *=* transverse relaxation time. ROIs were defined manually for germ (e.g., the shoot apical meristem, the radicle, the coleoptile, the plumule, and the scutellum). For all samples, *T*_2_ was calculated from ROIs that were drawn on the sagittal sides of the wheat. For the phantoms, a ROI in the form of a cylinder was drawn on the axial images. The transverse relaxation rate (R^2^) was obtained from the following equation: R^2^ = 1/*T*_2_ (s**^−^**^1^). Relaxivity (r_2_) was calculated as the slope of the linear regression line of a plot of R^2^ versus MRI contrast agent concentration [70].

Diffusion data were processed with a Paravision 360 v2.1. The results were consequently analyzed using the Bruker image sequence analysis tool. Signal intensity and standard deviation were derived from the internal fitting function ‘dtraceb’. Mono-exponential signal attenuation is I = A + C × exp (−b × ADC), where A in this equation represents absolute bias, C is the contrast term reflecting diffusion-related signal attenuation, b is the diffusion weighting factor, and ADC is the apparent diffusion coefficient. Five ROIs were selected from the embryo region for further calculations.

For the mapping of lipids in CSI images, the image slices were exported and analyzed in Bruker CSI Visualization Tool. To reconstruct the CSI images, a plug-in was used to select the area corresponding to the lipid signal. The hyperintense signal of the ROI was then calculated. The data were then exported to Origin Pro v. 8 software for further analysis.

The statistical analysis was carried out using a one-way analysis of variance (ANOVA) with OriginPro v. 8 (OriginLab, Northampton, MA, USA). This analysis aimed to compare relaxation times across five different ROIs (including the shoot apical meristem, the radicle, the coleoptile, the plumule, and the scutellum) in the embryo region, along with their corresponding relaxation times. For statistically significant results obtained from the one-way ANOVA, a Tukey post hoc analysis was performed as a multiple comparison of means test to evaluate the differences between individual groups. A significance level of *p* < 0.05 was used to determine statistical significance. To ensure the reliability of the results, the homogeneity of variance was evaluated using the Levene test. Groups with a *p*-value greater than 0.05 were considered to exhibit equal variance between them.

## 4. Conclusions

In this study, we introduced a biocompatible and non-invasive method for tracking the PET NPs in wheat seeds using fSPIONs as MRI contrast agents. The PET–fSPIONs were effectively visualized within wheat seeds, particularly in key developmental regions such as the shoot apical meristem, radicle, coleoptile, plumule, and scutellum. The successful attachment of PET NPs to fSPIONs was confirmed by comprehensive characterization, including TEM, DLS, and FTIR. The relaxivity measurements demonstrated that PET–fSPIONs are effective as MRI contrast agents, indicating their capacity to enhance image contrast in wheat seeds. Furthermore, SEM confirmed the presence and specific localization of PET NPs within the wheat embryo, thereby providing additional validation of the µMRI findings. DW-MRI and CSI revealed significant microstructural and biochemical alterations in the seeds treated with PET. The presence of PET–fSPIONs resulted in restricted diffusion and an increased lipid content due to exposure to PET NPs.

In conclusion, this study provides a valuable tool for future research into the environmental impact of plastic pollution and offers new perspectives on the interactions between nanoplastics and biological organisms.

## Data Availability

All relevant data are within the paper and its Supporting Information Files.

## Supplementary Materials

The following supporting information can be downloaded at: https://www.mdpi.com/article/10.3390/molecules29184380/s1, Figure S1: Particle size distribution curve obtained from TEM images for PET NPs and fSPION; Figure S2: SEM images showing the localization of PET-fSPION in wheat seeds.
