# Supplementary material for: Magnetic Resonance Imaging-Based Monitoring of the Accumulation of Polyethylene Terephthalate Nanoplastics
Source: Molecules. 2024 Sep 14;29(18):4380. doi: 10.3390/molecules29184380 (PMC11434537; doi:10.3390/molecules29184380)
Supplement: Supplementary file 1 [file molecules-29-04380-s001.zip › molecules-3132193-supplementary.pdf]

Supplementary information:

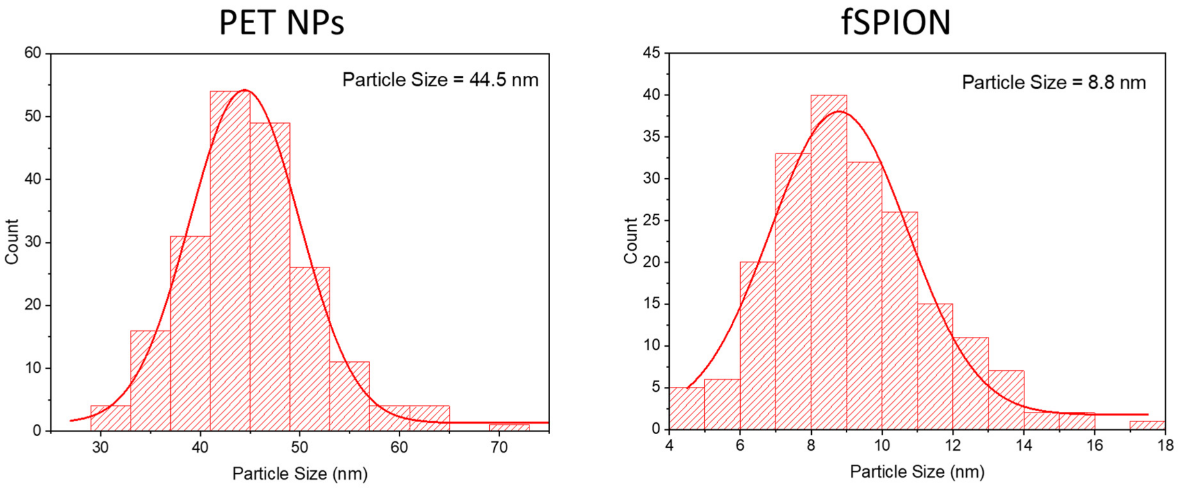

Figure S1. Particle size distribution curve obtained from TEM images for PET NPs and fSPION.

The size of these particles was estimated using ImageJ software. Nearly 300 particles from various TEM images were considered for plotting the histogram which is shown in Fig. S1 for PET NPs and fSPION samples. The histogram is fitted with the log-normal distributioncurve (red line) that fits with the mean size of 44.5 nm with a size distribution for the PET NPs sample and 8.8 nm with a size distribution for the fSPION sample.

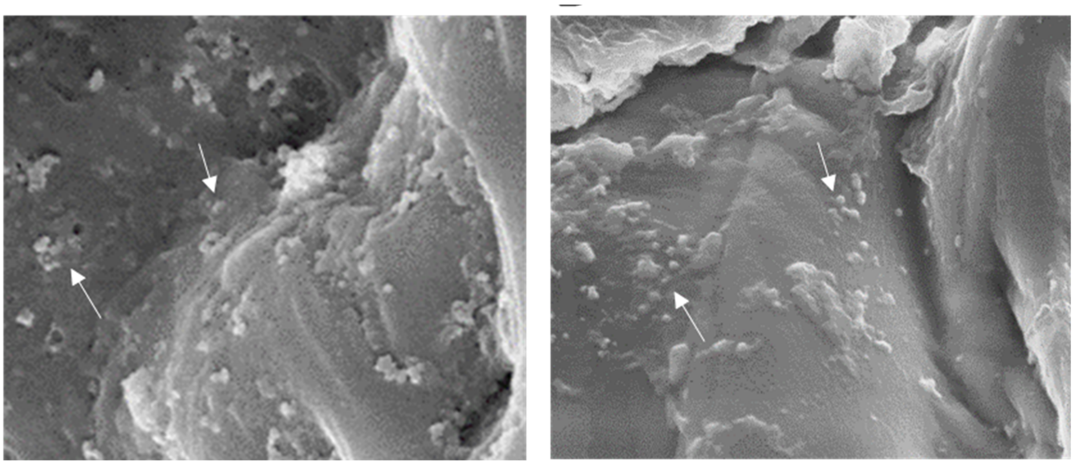

Figure S2. SEM images showing the localization of PET NPs (left) and PET-fSPION (right) in embryo region of wheat seeds.

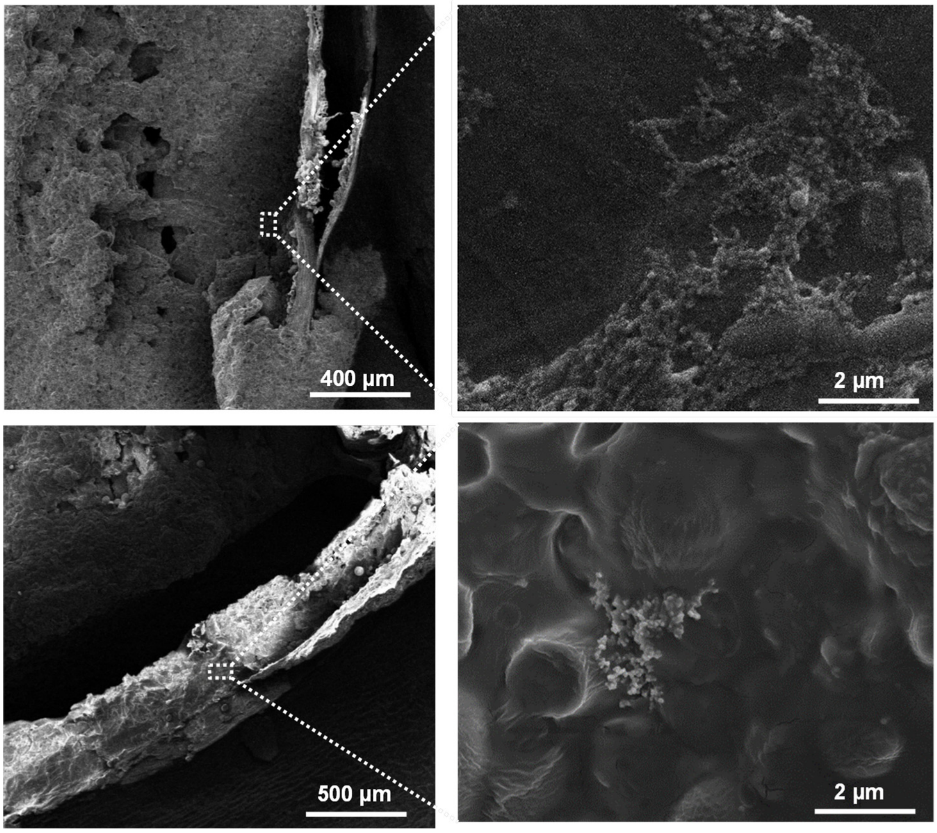

Figure S3. SEM images showing the localization of PET-fSPION in wheat seeds. Seeds were treated with a suspension of PET-fSPION for 24 h. Aggregations of PET-fSPION NPs are visible. Insets provide a closer view of the areas within the white squares, highlighting the detailed interaction between the nanoparticles and embryo surface.
